# Coaxial Electrospun Nanofibers of Shikonin and Cresol as Antibacterial Wound Dressing

**DOI:** 10.3390/ph18111642

**Published:** 2025-10-30

**Authors:** Fatemah M. Alsulaihem, Abrar A. Bakr, Meshal K. Alnefaie, Manal A. Alshabibi, Abdullah A. Alshehri, Fahad A. Almughem, Samar A. Alsudir, Ali A. Alamer, Bayan Y. Alshehri, Dunia A. Alzahrani, Fadilah S. Aleanizy, Essam A. Tawfik

**Affiliations:** 1 Advanced Diagnostics and Therapeutics Technologies Institute, Health Sector, King Abdulaziz City for Science and Technology (KACST), Riyadh 11442, Saudi Arabia; 2 Department of Pharmaceutics, College of Pharmacy, King Saud University, Riyadh 11495, Saudi Arabia; 3 Bioengineering Institute, Health Sector, King Abdulaziz City for Science and Technology (KACST), Riyadh 11442, Saudi Arabia

**Keywords:** electrospinning, antibacterial, wound healing, coaxial nanofibers, shikonin, cresol

## Abstract

**Background/Objectives**: Skin wounds interrupt the natural anatomy and function of the skin. The body passes through four physiological phases to repair wounds after injury. Since the fibers are more closely related to the extracellular matrix structure, they can be used as scaffolds to accelerate wound closure. Shikonin is a botanical herbal remedy used as an anti-inflammatory agent and for its wound-healing characteristics. Cresols are known for their bactericidal and fungicidal properties, which promote their utilization as a disinfectant in soap. Therefore, this study aimed to formulate shikonin and cresol-loaded nanofibers for a dual wound-healing and antibacterial wound dressing in vitro. **Methods**: This study demonstrated the effectiveness of the drug-loaded nanofibers against diverse Gram-positive and Gram-negative bacteria using the minimum inhibitory concentration (MIC) and zone of inhibition assays. **Results**: Scanning electron microscopy images showed successful formulation of shikonin/cresol fibers with an average diameter of 772 ± 152 nm. The encapsulation efficiency and drug loading for the dual drug-loaded fibers were 44 ± 1% and 25 ± 1 µg/mg, respectively, for shikonin, and 38 ± 1% and 21 ± 0.5 µg/mg, respectively, for cresol, with a full release of both drugs achieved after 180 min. The combination of both compounds exhibited a safe concentration of ≤6 µg/mL, with cell viability of >50% in human dermal fibroblasts (HFF-1) after 24 h. The MIC results indicated that the combination was efficient as an antibacterial agent against Gram-positive bacteria at a safe concentration. The shikonin/cresol-loaded fibrous system showed an inhibition zone close to that of the control drugs, suggesting that the drugs have retained their antibacterial activity after electrospinning. **Conclusions**: This dual drug-loaded fiber system showed a high potential as an antibacterial wound dressing for skin infection injuries. However, in vivo studies are required to assess the safety and efficacy in an animal model of the dual drug-loaded fiber system.

## 1. Introduction

The skin is considered the most crucial organ in the body, and has an essential role in sensory function, heat regulation, and defense from the external environment [1]. Skin wounds are defined as any damage to the skin that interrupts its natural anatomy and function [2]. The body passes through four physiological phases to repair wounds after injury. It starts with hemostasis, inflammation, and proliferation, and ends with skin remodeling [3]. Further, time is a critical factor in the wound-healing process and serves as the basis for the medical classification of wounds into acute and chronic categories [4].

Acute wounds are wounds that destroy the integrity of soft tissue and close naturally by following timely and systematic development (between 4 and 6 weeks). Chronic wounds have a prolonged healing period due to the failure of the usual healing phases and the presence of local infection. Another factor in skin infections is the dysbiosis of the skin microbiota and the topographical structure of the cutaneous microenvironment, which might influence the wound-healing process negatively. And it might increase the risk of exposed wounds to bacterial colonization and proliferation [5]. In the early phases of a chronic wound, Gram-positive bacteria, mostly *Staphylococcus aureus* (*S. aureus*), appeared at the maximum. Nevertheless, in progressive stages, Gram-negative bacteria such as *Escherichia coli* (*E. coli*) and *Pseudomonas species* frequently exist [6].

Depending on the wound type, numerous treatment options are available at various costs [7]. The surgical treatment for wounds includes debridement, skin grafts, and skin flaps, while the non-surgical treatment for wounds includes dressings, topical formulations, and skin substitutes [8,9,10].

Electrospinning is a technique that utilizes an electrostatic field to generate ultrafine fibers. In this process, a polymer is dissolved in water or an organic solvent and released from a syringe at a constant/controllable rate by the syringe pump. Because of the electrostatic force applied, a separation of positive and negative charges happens within the liquid, and charges of the same sign as the needle’s polarity move toward the surface, resulting in the creation of a charged polymer droplet at the needle’s tip [11]. The fibers are widely applied in antimicrobial dressings, tissue engineering, wound healing, and drug delivery due to their large surface area, flexibility, ease of preparation, and tensile strength. Particularly, since the nanofibers are more closely related to the ECM structure, which plays a vital role in wound healing, they can be used as scaffolds to accelerate wound closure [12]. Monoaxial (single-layered), coaxial (double-layered), and side-by-side (layer-by-layer) electrospinning are different types of electrospinning techniques [13]. The utilization of coaxial electrospinning processes enables the co-delivery of multiple drugs and regulates their release rates [14].

Shikonin (Shi), a natural naphthoquinone extracted from *Lithospermum erythrorhizon*, is used as a traditional Chinese remedy [15]. Shi has pharmacological activities, including antimicrobial, antioxidant, wound healing, anti-cancer, and anti-inflammatory activities [16,17]. Han et al. effectively encapsulated Shi in poly(ε-caprolactone) (PCL)/poly(trimethylene carbonate) (PTMC) nanofibers using the electrospinning method [18]. Arampatzis et al. developed novel Alkannin and Shi (A/S)-loaded polymeric nanofibers containing cellulose acetate (CA) or PCL. The developed nanofibers displayed antibacterial characteristics against *S. aureus* and *Staphylococcus epidermidis* (*S. epidermidis*) [19]. In another study by the same research group, electrospinning of polyhydroxybutyrate (PHB) was used to load A/S. A/S Shi-loaded PHB nanofibers have antibacterial activity against *S. epidermidis* and *S. aureus* [20].

Cresol (Cre) is a mixture of the three isomers of methylphenol, 2-methylphenol (o-cresol), 3-methylphenol (m-cresol), and 4-methylphenol (p-cresol). These isomers are produced as by-products of the steel industry during the pyrolysis procedure of mineral coal. They are used as an imperative intermediate in the synthesis of numerous materials in the chemical, food, and pharmaceutical industries. A mixture of isomers has also been marketed as disinfectants for domestic and veterinary use [21]. The antimicrobial activity of Cre was similar to that of phenol, but slightly higher. It is moderately active against Gram-positive bacteria and less active against Gram-negative bacteria. Cre has no significant activity against bacterial spores [22]. In addition, in pharmaceutical formulations intended for intramuscular, intradermal, and subcutaneous injections, Cre is used as an antimicrobial preservative at a concentration of 0.15–0.3%. It is also utilized as a preservative in some topical formulations and as a disinfectant [22].

Antimicrobial resistance (AMR) is a significant public health concern that impedes the effective prevention and treatment of an ever-increasing range of infections that are no longer vulnerable to standard medicines. Therefore, the development of novel antibacterial formulations is encouraged [23]. Hence, the combination of Cre and Shi in coaxial electrospun fibers should improve the treatment of infected wound injuries and enhance patient outcomes. Shi is used as a topical wound-healing and antibacterial medicinal product [24], while Cre will be utilized as an antimicrobial agent. The novelty of this work lies in its evaluation of dual-drug-loaded coaxial fibers, which combine Shi and Cre to serve as an antibacterial wound dressing. This dressing can inhibit bacteria and promote wound healing by enabling rapid and accelerated drug release at the wound site. PVP is chosen for its biocompatibility and solubility in both water and organic solvents [25].

## 2. Results and Discussions

### 2.1. Morphology Assessment of the Drug-Loaded Coaxial Nanofibers

**Figure 1:**
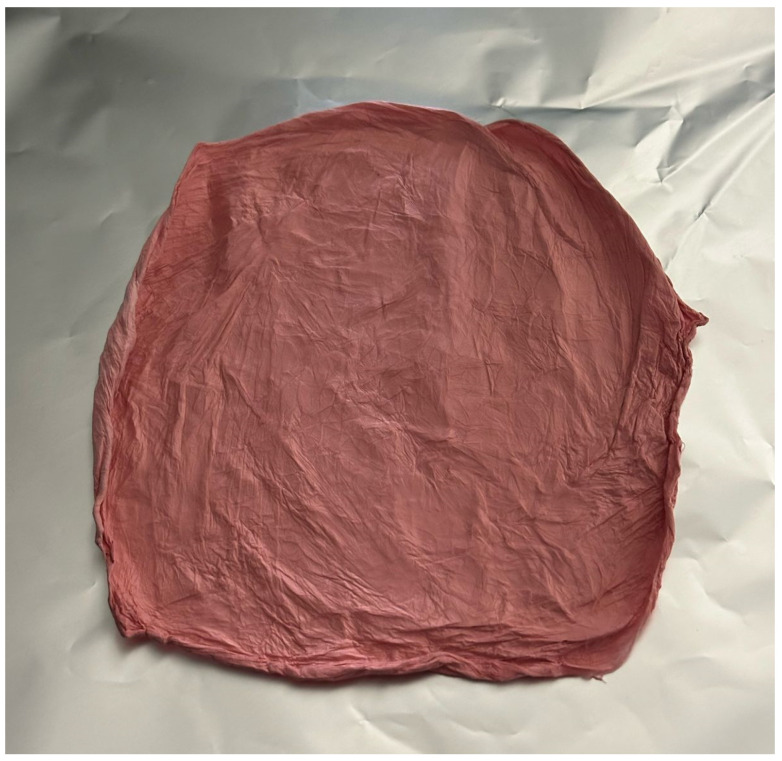
Shi/Cre-loaded fiber formulation after the electrospinning process.

**Figure 2:**
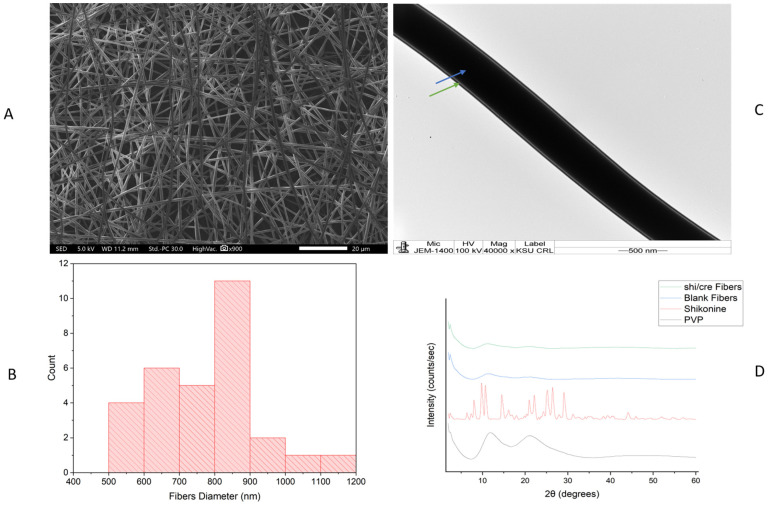
(**A**) SEM image showing the morphology of the Shi/Cre-loaded nanofibers as smooth, non-beaded, and non-porous surfaces. (**B**) Bar graph of the Shi/Cre-loaded fibers’ diameter distribution, indicating the average diameter of the nanofibers as 772 ± 152 nm. (**C**) TEM image of the Shi/Cre-loaded coaxial nanofibers, showing two distinctive layers, with the blue arrow pointing towards the inner layer containing the Cre, and the green arrow pointing towards the outer layer containing the Shi. (**D**) XRD patterns of PVP polymer, Shi, blank, and Shi/Cre-loaded coaxial fibers revealed that Shi was in the crystalline form (the presence of characteristic peaks) before being electrospun, while the PVP, blank, and Shi/Cre-loaded fibers were in the amorphous form (broad halo pattern), suggesting the molecular dispersion of Shi due to the electrospinning technique.

The drug-loaded coaxial fibers were successfully prepared following modified electrospinning parameters [26]. Figure 1 Shows Shi/Cre-l-loaded fibers produced by electrospinning. The figure shows the formulation after electrospinning, which indicates successful fiber formation. The color was pink because of the color of Shi. The SEM image in Figure 2A shows that the morphological surfaces of the nanofibers were smooth, non-beaded, and non-porous, indicating a successful electrospinning preparation method. The average diameter of this coaxial system was estimated as 772 ± 152 nm Figure 2B. These successful preparatory criteria were also observed for PVP coaxial fibers in previous studies [14,26,27]. The Shi/Cre-loaded fibers were also well assessed under the TEM. The image illustrated two distinct layers, representing the inner and outer layers of the core-shell nanofibrous system, as demonstrated in Figure 2C. This was also observed in the coaxial system of previous studies [14,26,27].

### 2.2. X-Ray Diffraction (XRD) Assessment

XRD is an analytical technique utilized to assess the drug’s crystallinity and to evaluate the amorphous nature of the solid dispersion. Figure 2D demonstrates the XRD pattern of PVP as a broad-halo peak due to the amorphous state of the PVP polymer, with no crystallinity peaks predicted to be detected, along with the blank PVP fibers [27]. The XRD pattern of Shi displayed a crystalline diffractogram via the existence of numerous intense Bragg reflection peaks. The pattern of Shi exhibited an order of intense Bragg reflections at 2θ: 6°, 8°, 9°, 10°, 14°, 16°, 21°, 22°, 24°, 26°, 29°, 31°, and 44°, which was in agreement with the Fu et al. XRD results for Shi [28]. The XRD spectrum displayed the fact that the highly intense peaks of Shi disappeared in the drug-loaded fibers, which demonstrated a broad halo. This suggests that the electrospinning technique allowed for the molecular transformation of the loaded drug, similar to the previous electrospun fiber studies of Aburayan et al. [12] and Alshaya et al. [26], which used halicin-loaded PVP fibers, and nifedipine and atorvastatin calcium-added PVP fiber systems, respectively. The XRD analysis for Cre was not performed owing to the presence of this drug in liquid form, and the XRD is an analytical method used to determine the solid crystal structures of a sample [29].

### 2.3. Drug Loading (DL) and Encapsulation Efficiency (EE) % Determination

**Figure 3:**
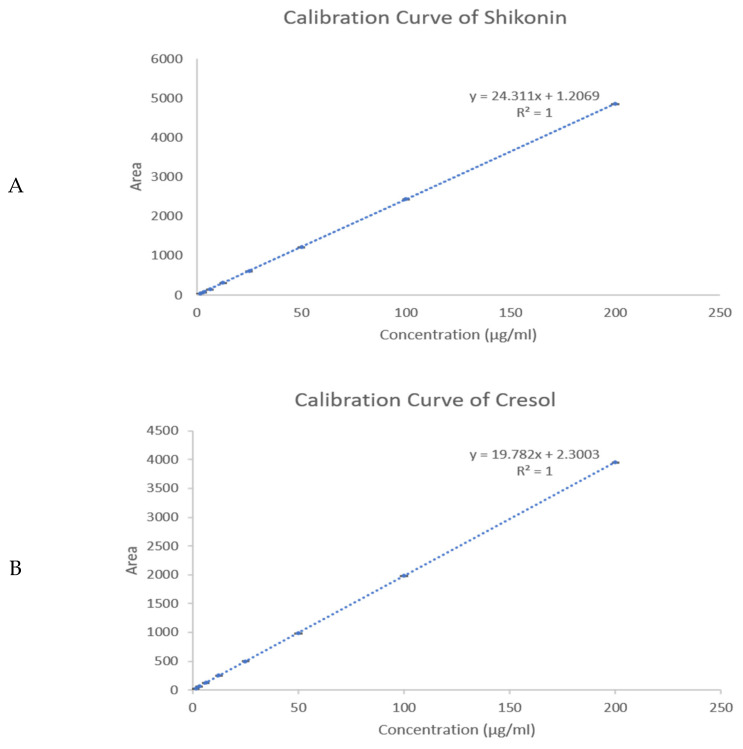
The calibration curve of (**A**) Shi and (**B**) Cre shows an excellent linearity of the developed HPLC method (with a concentration range from 1.5 to 200 μg/mL).

The EE% and DL of the Shi/Cre-loaded fibers were determined using the developed HPLC method utilizing the calibration curve, as shown in Figure 3. The average EE% and DL of the Shi/Cre nanofibers were found to be 44 ± 1% and 25 ± 1 µg/mg, respectively, for Shi, and 38 ± 1% and 21 ± 0.5 µg/mg, respectively, for Cre. The low EE% of Shi and Cre could be attributed to the dispersion of the remaining drug on the surface of the fiber as a result of the phase separation between the polymer and drugs, especially since PVP is amphiphilic, which makes it the most suitable polymer to dissolve the lipophilic drugs (i.e., Shi and Cre), while the hydrophilic properties of the PVP will allow for the immediate release of the loaded drugs. Although the efficiency was modest, the incorporated fraction was adequate to achieve the intended fast release and antibacterial activity. There is poor solubility of these lipophilic drugs in the polymeric solution, where the drug molecules may aggregate on the surface of the fiber. Thus, a good match of hydrophilic and hydrophobic properties between drugs and polymers is crucial for blending in electrospinning [30]. Han et al. have also shown a high solubility of Shi in the polymer PCL/PTMC and solvent DCM/DMF solution, suggesting that Shi’s compatibility with an organic polymer solution system might improve the EE% [18]. In addition, Kontogiannopoulos et al. have shown that the polymer with the highest total drug entrapment was PLLA with fibers incorporating an alkannin/Shi A/S mixture [31]. However, due to the flexibility of the nanofiber system, a section of fibers could be cut in correspondence with the targeted dose required.

### 2.4. In Vitro Drug Release Assessment

**Figure 4:**
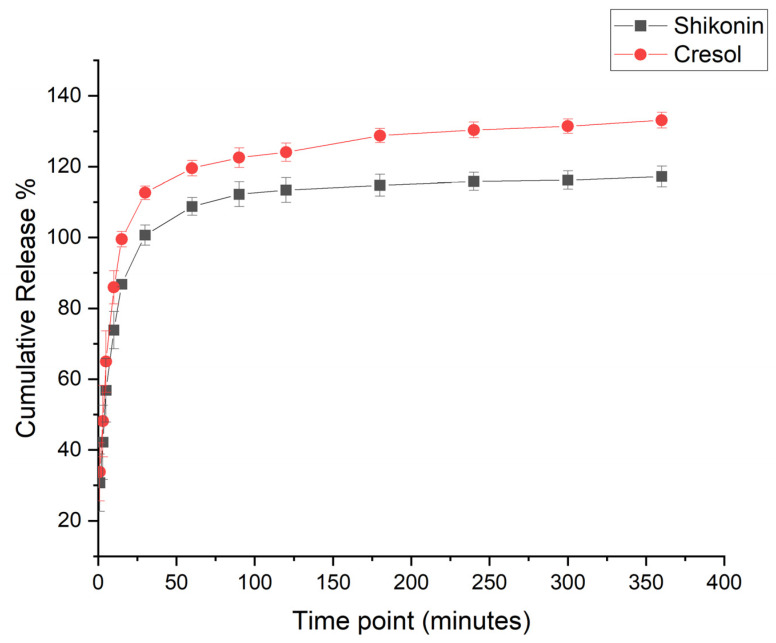
In vitro drug release profile of Shi/Cre-loaded coaxial fibers showing a burst release profile after 10 min, followed by a full drug release after 180 min.

The release profile of the Shi/Cre-loaded coaxial system was achieved by utilizing a release medium of 50% pre-warmed PBS (pH 7), 45% Acetonitrile, and 5% Tween 80 to overcome the low solubility of both drugs. The released amount of Shi and Cre at the first 10 min was 73% and 85%, respectively, reaching a complete drug release after 180 min, as shown in Figure 4. This outcome was anticipated because of the hydrophilic nature of the PVP polymer, which accelerated the dissolution and disintegration of the fibrous system. Also, due to the large surface area per unit volume of the nanofiber and the molecular transformation of the loaded drug (which was confirmed by the XRD analysis), the release of drugs was enhanced. The in vitro release study can be summarized as follows: diffusion drug release that was observed at the first 10 min (i.e., burst drug release), followed by polymer degradation, which led to the entire drug release at about 180 min. This accelerated release profile was in agreement with Aburayan et al. [12] and Sriyanti et al. [32], who assessed the release of halicin-loaded PVP fibers, and α-mangostin-loaded PVP fibers systems, respectively. Figure 4 also showed that the drug-loaded coaxial system had more than 100% release. This finding could be attributed to drug distribution variation within the nanofibers, which will require further investigation.

### 2.5. In Vitro Cell Viability Assessment

**Figure 5:**
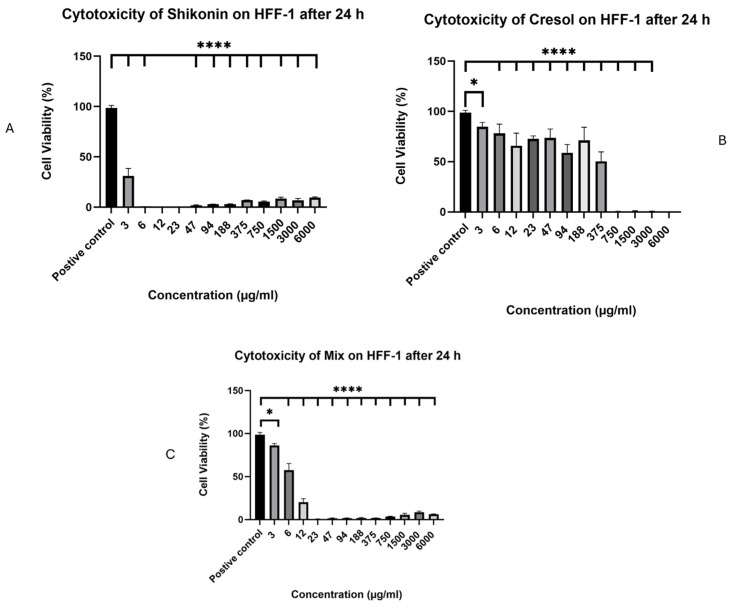
Cell viability of Shi (**A**), Cre (**B**), and Shi/Cre in a 1:1 combination (**C**) after 24 h of exposure to HFF-1 cells from 6000 to 3 µg/mL. The results displayed that Shi is unsafe if used alone, while Cre is safe up to 188 µg/mL. The combination enhanced the safety of Shi only up to 6 µg/mL. Statistical analysis was made using the One-Way ANOVA test (* *p* < 0.5, and **** *p* < 0.0001) [36].

The in vitro cell viability evaluation of Shi, Cre, and the combination (in 1:1) was conducted against a human skin fibroblast cell line (HFF-1). The results showed that Shi would be toxic if used at three µg/mL alone, with cell viability of ≤30% after 24 h of cell exposure (Figure 5A). This result was similar to a study published in 2015 by Fan et al., who reported that three µg/mL of Shi is toxic in human dermal fibroblasts and suppresses cell proliferation [33].

Conversely, Cre displayed a safer profile than Shi and may be safe at ≤188 µg/mL (Figure 5B). The lower toxicity of Cre might be due to its antioxidant effect. A study published in 2002 by Yeung et al. stated that three Cre isomers may exert antioxidative effects and, consequently, have the potential to prevent oxidative stress in the pulp and periapical tissues [34]. Despite its antioxidant effects, a study published in 2014 by Chang et al. reported that p-Cre may cause thrombosis and atherosclerosis in patients suffering from Cre intoxication and uremia. This could be because of the generation of ROS, endothelial and mononuclear cell damage, and the formation of inflammation and atherosclerosis-related molecules [35].

The results of the Shi and Cre combination exhibited safe concentrations of up to 6 µg/mL with cell viability above 50% after 24 h of cell exposure (Figure 5C). These findings suggest that Shi would be toxic if used alone at ≥6 µg/mL, but if used with Cre, its toxicity may be reduced.

### 2.6. Antibacterial Minimum Inhibitory Concentration (MIC) Assessment

The MIC was used to assess the effectiveness of Shi and Cre separately and in combination at a 1:1 ratio against Gram-positive bacteria, including *S. aureus* (ATCC 29213) and methicillin-resistant *S. aureus (MRSA*; ATCC 43300). The Gram-negative bacteria included *Pseudomonas aeruginosa* (*P. aeruginosa*; ATCC 27853), *Escherichia coli* (*E. coli*; ATCC 25922), and *Acinetobacter baumannii* (*A. baumannii*; ATCC BAA 747). Clinical isolates of the Gram-negative resistant bacterial strains were also evaluated, including *P. aeruginosa* (isolate 7067), *E. coli* (isolate 1060), and *A. baumannii* (isolate 3034). The MIC was measured at concentrations ranging from 6000 to 3 µg/mL dissolved in DMSO. The DMSO showed no effect on Gram-positive bacteria, while it had an impact against the Gram-negative bacteria at the highest concentration (i.e., 6000 µg/mL). The presence of turbidity indicated bacterial growth, and the lowest concentration with no visible growth (i.e., clear) was considered the MIC.

The MICs of Shi, Cre, and the 1:1 combination showed more susceptibility to Gram-positive bacteria than Gram-negative bacteria. The MICs of Shi against *S. aureus* and *MRSA* were 3 and 6 μg/mL, respectively. The MICs of the 1:1 combination for *S. aureus* and *MRSA* were 3 μg/mL, as summarized in Table 1.

A study by Shu et al. has investigated a Shi-loaded liposome formulation on burn-infected wounds. The study revealed that the MRSA strain was inhibited at a lower concentration of 64 μg/mL compared to the free Shi at 128 μg/mL. This effect was linked to rupture of the bacterial cell wall, as observed through transmission electron microscope (TEM) analysis. Furthermore, they have shown an increase in electrical conductivity, β-galactosidase, and alkaline phosphatase (AKP) levels, with a decrease in lactate dehydrogenase (LDH) and intracellular protein content levels [37]. Another study reported similar results for Shi against MRSA, with TEM images showing disruption of the cytoplasmic membrane, leading to cell lysis and leakage of intracellular materials, resulting in cell death. These findings might indicate interference with bacterial metabolism and cell structure disruption, leading to death [38].

A study by Qiu et al. found that the MICs of Shi for *S. aureus*, *P. aeruginosa*, *E. coli*, and *B. subtilis* were 3.1, 9.6, 3.9, and 3.1 μg/mL, respectively [39]. It was also reported that Shi and its derivatives have shown to be active against Gram-positive bacteria such as *S. aureus*, *E. faecium*, *and Bacillus subtilis* (*B. subtilis)* at MICs ranging from 0.30 to 6.25 mg/mL. In contrast, they are inactive against Gram-negative bacteria such as *E. coli*, *P. aeruginosa*, and *Micrococcus luteus* [40]. Shi has better antibacterial activity against *S. aureus*, and this could be linked to its chemical structure (i.e., naphthoquinone compound) since quinone has a potent antibacterial activity [41]. In contrast, Cre showed higher MIC results of 1500 μg/mL against Gram-positive bacteria and 750 μg/mL against Gram-negative bacteria. The MIC of Cre was within two-fold of that reported by Shin et al. The study demonstrated the essential oil fraction of *Ostericum koreanum* that was analyzed by gas chromatography coupled with mass spectrometry (GC-MS). The GC-MS analysis revealed that 17.99% of the oil component is *p*-cresol, which showed the most inhibitory effects, with MICs ranging from 2 to 4 mg/mL against *Salmonella enteritidis* (*S. enteritidis*) and *Salmonella typhimurium* (*S. typhimurium*), which are typical causes of food-borne diseases in humans and animals [42]. The phenolic germicides’ mechanism of action suggests that their efficacy might be due to physical damage to bacterial cell membranes [43]. Overall, these findings indicate that Shi and its combination might be more potent than Cre alone. Further research is necessary to explore the variations in MIC values reported in the literature and to understand the mechanism of action involved in the antibacterial activity of Shi and Cre on bacteria.

### 2.7. Antibacterial Zone of Inhibition Assessment

**Figure 6:**
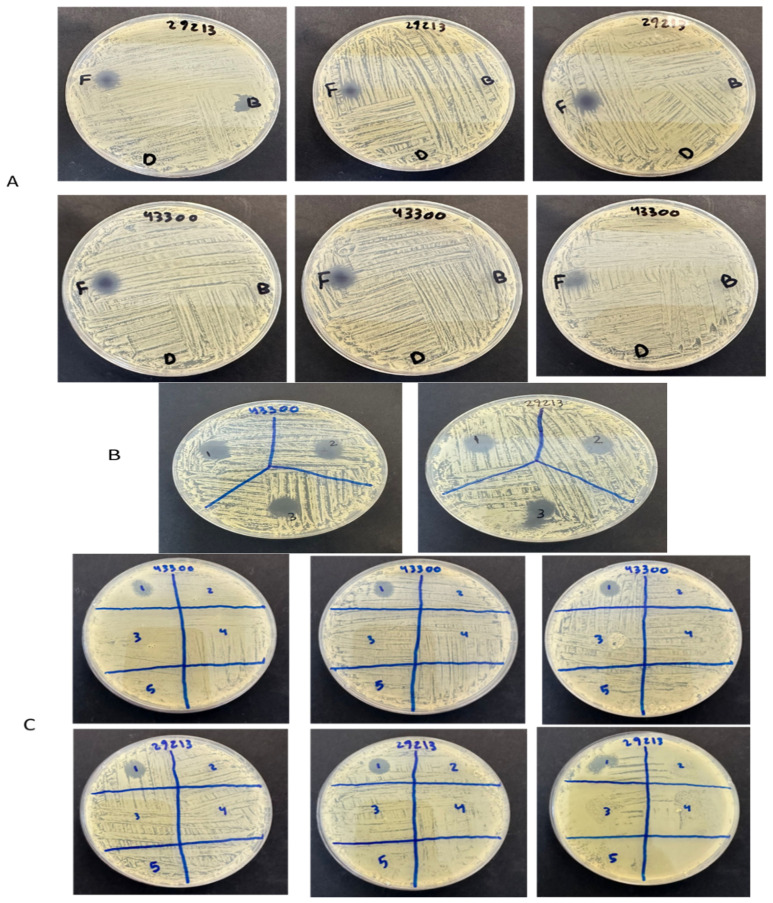
(**A**) Zone of inhibition of the Shi/Cre nanofibers against bacteria strains *S. aureus*—ATCC 29213 and *MRSA*—ATCC 43300. (F) is Shi/Cre fiber, (B) is blank PVP, and (0) is no drug. (**B**) Zone of inhibition of the control drugs Shi/Cre (in a similar dose to the Shi/Cre nanofibers) against bacteria strains *S. aureus*—ATCC 29213 and *MRSA*—ATCC 43300. (**C**) Zone of inhibition of Vancomycin against bacteria strains *S. aureus*—ATCC 29213 and *MRSA*—ATCC 43300, where 1, 8 μg/mL; 2, 4 μg/mL; 3, 2 μg/mL; 4, 1 μg/mL; 5, 0.5 μg/mL.

The antibacterial efficacy of the dual Shi/Cre-loaded fibers and the free drugs of Shi/Cre was evaluated using the zone of inhibition assay against the Gram-positive bacteria strains only, i.e., *S. aureus* (ATCC 29213) and *MRSA* (ATCC 43300). Blank fibers and vancomycin were used as negative and positive controls, respectively. The zone of inhibition for the drug-loaded fibers and control drugs was observed at varying diameters (Figure 6). The zone-of-inhibition diameter of Shi/Cre fibers was approximately 10 mm against both *S. aureus* (ATCC 29213) and MRSA (ATCC 43300), as shown in Table 2. Additionally, the zone of inhibition for the control drugs Shi/Cr at a similar dose to the drug-loaded fibers was around 12 mm against *S. aureus* (ATCC 29213) and 13 mm against MRSA (ATCC 43300).

The Shi/Cre fibers exhibited a zone of inhibition very similar to that of the free drugs Shi/Cre, which could confirm the retention of the antibacterial effect of the drugs after electrospinning, particularly Shi. The slight reduction in the diameter of the drug-loaded nanofibers might be attributed to the variation (SD) in the DL of the drug-loaded nanofibers. The blank nanofibers, as a negative control, showed no bacterial inhibition, while the vancomycin, as a positive control, demonstrated a zone of inhibition of 8 mm against *S. aureus* (ATCC 29213) and 9 mm against MRSA (ATCC 43300) at a concentration of 8 μg/mL (Figure 6C).

A previous study by Arampatzis et al. demonstrated that Shi loaded into PHB nanofibers had an antibacterial activity, with the zone of inhibition observed at a diameter of 11.81 mm and 13.72 mm for *S. epidermidis* and *S. aureus*, respectively [20]. Moreover, a study by Han et al. demonstrated the antibacterial activity of Shi-loaded PCL/PTMC nanofibers, in which the 5 wt% Shi-loaded specimen gave a diameter of 21.3 mm for *S. aureus* and 16.9 mm for *E. coli* [18].

Overall, the ability of the dual drug-loaded nanofiber system to retain its antibacterial activity holds great promise for application against Gram-positive infected wounds, particularly since concentrations of ≤6 μg/mL might be safe for daily use. Furthermore, in vivo studies on infected wounds are required to evaluate the wound closure ability of the developed nanofibrous system.

## 3. Materials and Methods

### 3.1. Materials

Shi (molecular weight of 288.30 g/mol) was purchased from BOC Sciences (Shirley, NY, USA). Mixed Cre (molecular weight of 108.14 g/mol), Polyvinylpyrrolidone (PVP, average molecular weight ~1,300,000), DMSO, fetal bovine serum (FBS), penicillin, and streptomycin were all provided by the Sigma-Aldrich Chemical Company (St. Louis, MO, USA). Methanol, ethanol, and acetonitrile were all High-Performance Liquid Chromatography (HPLC) grade and were obtained from Fisher Scientific UK Ltd. (Loughborough, Leicestershire, UK) and from the American Type Culture Collection (ATCC) (Manassas, VA, USA). Both the referenced bacterial strains and the human skin fibroblast cell line (HFF-1, ATCC number SCRC-1041) were obtained. Mueller–Hinton agar (MHA) and Mueller–Hinton broth (MHB) were purchased from Scharlau (Barcelona, Spain) and prepared according to the manufacturer’s instructions. Distilled water used in the experiments was generated using the Milli-Q^®^ IQ 7005 Purification System (Millipore SAS, Molsheim, France). MTS reagent (Cell Titer 96 ^®^Aqueous One Solution Cell Proliferation Assay) was purchased from Promega (Southampton, UK). Dulbecco’s Modified Eagle Medium (DMEM) was purchased from PAN BIOTECH (Aidenbach, Germany). Phosphate-buffered saline (PBS) was purchased from Gibco, Thermo Fisher Scientific, Inc. (Waltham, MA, USA).

### 3.2. Drug-Loaded Coaxial PVP Fibers

A drug-loaded spinning solution was prepared in vitro using the Spraybase^®^ electrospinning system (Dublin, Ireland) by utilizing PVP and modifying the electrospinning parameters [26]. PVP was dissolved in absolute ethanol (4 mL) at a concentration of 8% (*w*/*v*). The spinning solution was stirred at ambient temperature for at least 60 min. After the complete dissolving of the PVP, 0.5% (*w*/*v*) Shi and 0.5% (*w*/*v*) Cre were separately added to the polymer solutions (i.e., into two separate vials) and stirred for another 60 min to achieve complete dissolving of the drugs into the polymer solution, and to avoid any possible interaction between both drugs; hence, they were in two separate layers. The coaxial needle was used with inner and outer diameters of 0.45 and 0.9 mm, respectively. The needle tip-to-collector distance was maintained at 15 cm, the flow rate was adjusted at 1 mL/hour, and the applied voltage was increased gradually and set between 8 and 10 kV to acquire a stable jet. Blank (drug-free) fibers were prepared similarly, but without the addition of any drug.

### 3.3. Scanning Electron Microscopy (SEM) Assessment

The fibers’ morphology was scanned and analyzed using SEM (JSM-IT500HR, Peabody, MA, USA). The samples were coated with platinum with a thickness of 3 nm by (JEC-3000F, JEOL, Peabody, MA, USA), which was used for coating processing, and the fibers were examined at an accelerating voltage of 5 kV. The fibers’ diameters were analyzed using the ImageJ software, 1.54g (National Institute of Health, Bethesda, MD, USA).

### 3.4. Transmission Electron Microscopy (TEM) Assessment

The core and shell layers of the coaxial fibers were differentiated by the TEM (JEM-1400 TEM, JEOL, Tokyo, Japan). During the electrospinning process, the fibers were collected directly onto a copper grid and studied at an accelerating voltage of 100 kV.

### 3.5. X-Ray Powder Diffraction (XRD) Assessment

A Rigaku Miniflex 300/600 (Tokyo, Japan), equipped with a Cu Kα radiation source, an excitation voltage of 40 kV and a current of 15 mA, was utilized. The Shi, PVP, drug-loaded, and blank fibers were analyzed by placing the sample on the holder. The recording analysis was taken from a 2θ range of 2° to 60° at a scan speed of 5°/min.

### 3.6. Quantification Using High-Performance Liquid Chromatography (HPLC) Determination

The HPLC analysis was performed on a 1260 Infinity II HPLC system (Agilent, Santa Clara, CA, USA). The system contains a two-solvent gradient flexible pump, an autosampler, and a UV detector [26]. The chromatographic separation of Shi and Cre was performed via an HPLC Column Poroshe11 120 EC-C18 4 μm (4.6 × 150 mm). The mobile phase system consisted of A 40% [0.01 M potassium phosphate (monobasic) in water (pH = 3.3)] and B 60% methanol. Mobile phase B (methanol) was started at 60% for 5 min and increased to 70% at a linear gradient for the next 5 min, 75% for the next 5 min, and 60% for the last 1 min, for equilibrium. The total run time was 16 min, and the flow rate was 1.0 mL/min. The UV detection wavelength was adjusted to 275 nm [44,45].

Shi and Cre stock solutions were initially dissolved separately in methanol. Shi was then mixed with Cre, and the mixture was dissolved in the mobile phase to make serial dilutions of both drugs at equal concentrations. Shi and Cre were detected at retention times of Rt = 14 min and 2 min, respectively. The calibration curves for both drugs were in equal concentrations. Shi and Cre were detected at retention times of Rt = 14 min and 2 min, respectively. Calibration curves of both drugs were constructed using a series of concentrations ranging between 1.5 and 200 μg/mL.

### 3.7. Drug Loading (DL) and Encapsulation Efficiency (EE) % Determination

Measuring the DL and EE% of the drug-loaded nanofibers was carried out by dissolving fibers (weighing 5 ± 0.5 mg) in 10 mL methanol, and was kept stirring for 24 h in a dark area because Shi and Cre are both photosensitive drugs [22,46]. The HPLC method was used to analyze drug content in the fibers, where the DL and EE% values were determined according to the following equations:

(1) $$DL=\frac{Entrapped\;drug\;amount}{Weight\;of\;the\;fibers}$$

(2) $$EE\%=\frac{Actual\;drug\;amount}{Theoretical\;drug\;amount}\times 100$$

The theoretical drug content was calculated based on the weight of the solid materials (polymers and drugs) that were intended for electrospinning. The results are presented as the average ± standard deviation (SD) of four replicates.

### 3.8. In Vitro Drug Release Assessment

Measuring the drug release was carried out by putting approximately 30 mg of the drug-loaded fibers into glass vials with 15 mL of release medium composed of 50% pre-warmed PBS (pH 7), 45% Acetonitrile, and 5% (Tween 80) to facilitate the solubility of both drugs. Then, the vials were held in a thermostatic shaking incubator (Excella E24 Incubator Shaker Series, New Brunswick Scientific Co., Enfield, CT, USA) at 37 ± 0.1 °C and 100 rpm. After 1, 3, 5, 10, 15, 30, 60,90, 120, 180, 240, and 300, 360 min, samples measuring 1 mL were withdrawn and replaced with equal volumes of the pre-warmed release medium. The cumulative release % was estimated as a function of time and was calculated according to the following equation.

(3) $$Cumulative\;Release\;\%=\frac{Cumulative\;drug\;amount}{Therotical\;drug\;amount}\times 100$$

### 3.9. In Vitro Cell Viability Assessment

The in vitro cell viability evaluation of Shi, Cre, and their 1:1 combination was conducted against HFF-1 human skin fibroblasts using MTS reagent. The 3-(4,5-dimethylthiazol-2-yl)-5-(3-carboxymethoxyphenyl)-2-(4sulfophenyl)-2H-tetrazolium inner salt (MTS) assay is an altered, better version of the MTT assay [47]. The assay was conducted after incubating the drugs with the HFF-1 cells for 24 h, as the intended application would be daily. DMEM was used to culture the human cells, and was complemented with 10% (*v*/*v*) FBS, penicillin 100 U/mL, and streptomycin 100 gm/mL. The trypsin and trypan blue exclusion test was used to harvest and count the HFF-1 cells, followed by seeding into 96-well plates at a seeding density of 1 × 10^4^ cells/well. Cells were incubated in a cell culture incubator at 5% CO_2_ and 37 °C overnight. The tested compounds of 100 µL of increasing concentration (from 6000 to 3 µg/mL) were then added to the cell lines for 24 h. The cells utilized as the positive control were exposed to 0.1% Triton X-100, while the negative control was cells that were exposed to DMEM only. After the incubation, samples were aspirated from the wells and washed with sterile PBS at pH 7. Next, 100 µL of fresh complete DMEM was added to each well, followed by the addition of 20 µL of the MTS reagent. Lastly, cells were incubated for 3 h at 37 °C and 5% CO_2_. To measure the absorbance of the MTS solution, a microplate reader Cytation 3 (BIOTEK instruments Inc., Winooski, VT, USA) was used at a wavelength of 490 nm. Then, the cell viability % was measured utilizing the following equation:

(4) $$Cell\;Viability\;\%=\frac{\left(S-T\right)}{\left(H-T\right)}\times \;100$$

where S is the absorbance of cells subjected to the tested compound, T is the absorbance of cells subjected to Triton X-100 (positive control), and H is the absorbance of cells subjected to DMEM (negative control) [12,26].

### 3.10. Antibacterial Minimum Inhibitory Concentration (MIC) Assessment

The MIC assay was performed in vitro according to previously published methods and following the CLSI guidelines [12,48]. MIC was determined using the microbroth dilution method in 96-well microtiter plates, where 100 µL of MHB was dispensed into each well. Then, a serial dilution of Shi and Cre as a single or 1:1 combination at concentrations ranging from 6000 to 3 μg/mL was added into the 96-well plates in duplicate [49]. Gram-positive and Gram-negative bacterial inoculants were adjusted to 0.5 McFarland. And 100 µL was inoculated into the treated wells, achieving a final inoculum of 1 × 10^5^ CFU/mL. Positive control was bacteria only, and negative control was the medium-only group. The plates were incubated overnight at 37 ± 2 °C with a continuous shaking speed of 160 rpm. The lowest concentration with no visible growth was considered the MIC assay performed in duplicate.

### 3.11. Antibacterial Zone of Inhibition Assessment

The antibacterial effectiveness of the dual Shi- and Cre-loaded fibers was evaluated in vitro using the zone of inhibition assay against Gram-positive bacteria strains, including *S. aureus* (ATCC 29213) and MRSA (ATCC 43300). The Gram-negative bacteria were excluded because Shi and Cre were unaffected in the safe concentration, as confirmed by the previous in vitro cell viability and MIC assessments. The zone-of-inhibition assay was performed according to previously published methods [12].

An inoculum concentration of 1 × 10^5^ CFU/mL was spread onto the MHA surface. According to the MIC and DL outcomes, 0.12 mg of the drug-loaded fibers and the blank were weighed and then placed in MHA plates. Free drugs of Shi mixed with Cre, containing an equal concentration to that of the drug-loaded fibers, were used as an experimental control. The blank and drug-loaded fibers were evaluated against *S. aureus* (ATCC 29213) and MRSA (ATCC 43300) and incubated at 37 °C overnight. After that, the apparent diameters of ‘no growth’ were measured in mm. Additionally, vancomycin was also used as a control antibiotic.

### 3.12. Statistical Analysis

Drug release, antibacterial zone-of-inhibition experiments, and in vitro cell viability experiments were performed in three replicates, and the results are presented as the average ± SD. Statistical analysis was performed using OriginPro^®^ 2024 (OriginLab Corporation, Northampton, MA, USA) and Microsoft Excel 2019 software. For the in vitro cell viability experiments, statistical analysis was made using the One-Way ANOVA test (* *p* < 0.5, ** *p* < 0.01, *** *p* < 0.001, and **** *p* < 0.0001).

## 4. Conclusions

The biomedical use of the nanofibrous wound dressings holds great promise, owing to the advantages this system holds. To cure a skin infection wound, this study developed a fibrous biomaterial using a biocompatible, biodegradable and FDA-approved polymer, i.e., PVP, which could be applied as an anti-bacterial wound dressing. Shi/Cre-loaded fibers were successfully prepared and characterized using a coaxial electrospinning process. Characterization via SEM revealed a smooth, non-porous, and non-beaded morphology, while TEM analysis demonstrated the inner and outer layers. The EE% and DL for the Shi/Cre nanofibers were found to be 44 ± 1% and 25 ± 1 µg/mg, respectively, for Shi, and 38 ± 1% and 21 ± 0.5 µg/mg, respectively, for Cre, which indicates the successful loading of both drugs within this nanofibrous system. The in vitro drug release study exhibited a burst release of Shi (73%) and Cre (85%) released in the first 10 min, followed by a full drug release after 180 min. The in vitro cytotoxicity study of Shi, Cre, and the 1:1 combination indicated that the combination is safe at concentrations ≤ 6 µg/mL after 24 h exposure to human dermal fibroblasts. The antimicrobial MIC study demonstrated that Shi and the 1:1 combination were more effective than Cre against the Gram-positive bacteria. At the same time, Cre was more effective against the Gram-negative bacteria, except for the *A. baumannii*.

Our findings also indicated that the antibacterial activity of the drugs was retained after electrospinning upon the evaluation of the Shi/Cre-loaded fibers against the Gram-positive bacteria, particularly against *S. aureus*. The limitations found in this study are as follows. First, an in vivo assessment of an infected wound should be considered in the future to determine the efficacy of wound closure using this dual drug-loaded nanofiber system. Second, there was variability in the drug distribution within the dual drug-loaded nanofiber system. Third, the optimization of polymer composition and process parameters will be required to improve drug encapsulation in the future. Lastly, the stability of the fibers under storage conditions was not tested, and should be considered in future work. Overall, our study may provide a basis for the future clinical application of this Shi/Cre-loaded nanofibrous system as a promising wound dressing for Gram-positive skin infections against *S. aureus*.

## Figures and Tables

**Table 1:** The MIC assay against *S. aureus* (ATCC 29213), *MRSA* (ATCC 43300), *E. coli* (ATCC 25922), *E. coli* (isolate 1060), *A. baumannii* (ATCC BAA 747), *A. baumannii* (isolate 3034), *P. aeruginosa* (ATCC 27853), and *P. aeruginosa* (isolate 7067). Strains were treated with Shi and Cre alone and in a 1:1 combination at concentrations of 6000 to 3 μg/mL. Supplementary Figures S1–S9 display the MIC results.

Strains of Bacteria	Shikonin (μg/mL)	Cresol (μg/mL)	Mix (μg/mL)
*S. aureus*—ATCC 29213	3	1500	3
*MRSA*—ATCC 43300	6	1500	3
*E. coli*—ATCC 25922	No inhibition	750	750
*E. coli*—isolate 1060	No inhibition	750	1500
*A. baumannii*—ATCC 747	No inhibition	750	750
*A. baumannii*—isolate 3034	1500	750	375
*P. aeruginosa*—ATCC 27853	1500	750	1500
*P. aeruginosa*—isolate 7067	1500	750	1500

**Table 2:** Zone of inhibition of Shi/Cre-loaded nanofibers against bacteria strains *S. aureus* (ATCC 29213) and *MRSA* (ATCC 43300).

Strains of Bacteria	Control Drugs (Shi/Cre)	Shi/Cre Fibers	Blank Fibers	Vancomycin
*S. aureus*—ATCC 29213	12 mm	10 mm	0 mm	8 mm
MRSA—ATCC 43300	13 mm	10 mm	0 mm	9 mm

## Data Availability

The original contributions presented in this study are included in the article/Supplementary Material. Further inquiries can be directed to the corresponding author(s).

## Supplementary Materials

The following supporting information can be downloaded at: https://www.mdpi.com/article/10.3390/ph18111642/s1. Figure S1: The 96-well plate figure represents the (MIC) of the blank plate for shi and the mix (shi mixed with cre 1:1) without any bacteria. Figure S2: The MIC test against *S. aureus* (ATCC 29213). The green square was the MIC, which showed a clear well. Shi and the combination were at 3 μg/mL, and cre was at 1500 μg/mL. Figure S3: The MIC test against MRSA (ATCC 43300) as a clinical isolate. The green square was the MIC, which showed a clear well. Shi was at 6 μg/mL, cre was at 1500 μg/mL, and the combination was at 3 μg/mL. Figure S4: The MIC test against *E. coli* (ATCC 25922). The green square was the MIC, which showed a clear well. Shi has no inhibition, the cre was 750 μg/mL, and the combination was 750 μg/mL. Figure S5: The MIC test against *E. coli* (isolate 1060) as a clinical isolate. The green square was the MIC, shi has no inhibition, cre was 750 μg/mL, and the combination was 1500 μg/mL. Figure S6: The MIC test against *A. baumannii* (ATCC BAA 747). The green square was the MIC, which showed a clear well. Shi has no inhibition, and the cre was 750 μg/mL, and the combination was 750 μg/mL. Figure S7: The (MIC) test against *A. baumannii* (isolate 3034). The green square was the MIC, which showed a clear well. shi was 1500 μg/mL, cre was 750 μg/mL, and the combination was 375 μg/mL. Figure S8: The MIC test against *P. aeruginosa* (ATCC 27853). The green square was the MIC, which showed a clear well. Shi was 1500 μg/mL, cre was at 750 μg/mL. And the combination was at 1500 μg/mL. Figure S9: The MIC test against *P. aeruginosa* (isolate 7067) as a clinical isolate. The green square was the MIC, which showed a clear well. Shi was 1500 μg/mL, cre was at 750 μg/mL, and the combination was 1500 μg/mL.

## References

[1] Yang S., Li X., Liu P., Zhang M., Wang C., Zhang B. (2020). Multifunctional Chitosan/Polycaprolactone Nanofiber Scaffolds with Varied Dual-Drug Release for Wound-Healing Applications. ACS Biomater. Sci. Eng..

[2] Yazarlu O., Iranshahi M., Kashani H.R.K., Reshadat S., Habtemariam S., Iranshahy M., Hasanpour M. (2021). Perspective on the application of medicinal plants and natural products in wound healing: A mechanistic review. Pharmacol. Res..

[3] Wilkinson H.N., Hardman M.J., Wilkinson H.N. (2020). Wound healing: Cellular mechanisms and pathological outcomes. Open Biol..

[4] Nourian Dehkordi A., Mirahmadi Babaheydari F., Chehelgerdi M., Raeisi Dehkordi S. (2019). Skin tissue engineering: Wound healing based on stem-cell-based therapeutic strategies. Stem Cell Res. Ther..

[5] Van Rensburg J.J., Lin H., Gao X., Toh E., Fortney K.R., Ellinger S., Zwickl B., Janowicz D.M., Katz B.P., Nelson D.E. (2015). The human skin microbiome associates with the outcome of and is influenced by bacterial infection. MBio.

[6] Negut I., Grumezescu V., Grumezescu A.M. (2018). Treatment strategies for infected wounds. Molecules.

[7] Mirhaj M., Labbaf S., Tavakoli M., Seifalian A.M. (2022). Emerging treatment strategies in wound care. Int. Wound J..

[8] Bellingeri A., Falciani F., Traspedini P., Moscatelli A., Russo A., Tino G., Chiari P., Peghetti A. (2016). Effect of a wound cleansing solution on wound bed preparation and inflammation in chronic wounds: A single-blind RCT. J. Wound Care.

[9] Kolimi P., Narala S., Nyavanandi D., Youssef A.A.A., Dudhipala N. (2022). Innovative Treatment Strategies to Accelerate Wound Healing: Trajectory and Recent Advancements. Cells.

[10] Gushiken L.F.S., Beserra F.P., Bastos J.K., Jackson C.J., Pellizzon C.H. (2021). Cutaneous wound healing: An update from physiopathology to current therapies. Life.

[11] Xue J., Wu T., Dai Y., Xia Y., States U., States U. (2019). Electrospinning and Electrospun Nanofibers. Methods Mater. Appl..

[12] Aburayan W.S., Booq R.Y., BinSaleh N.S., Alfassam H.A., Bakr A.A., Bukhary H.A., Alyamani E.J., Tawfik E.A. (2020). The delivery of the novel drug ‘halicin’ using electrospun fibers for the treatment of pressure ulcer against pathogenic bacteria. Pharmaceutics.

[13] Farhaj S., Conway B.R., Ghori M.U. (2023). Nanofibres in Drug Delivery Applications. Fibers.

[14] Alkahtani M.E., Aodah A.H., Abu Asab O.A., Basit A.W., Orlu M., Tawfik E.A. (2021). Fabrication and characterization of fast-dissolving films containing escitalopram/quetiapine for the treatment of major depressive disorder. Pharmaceutics.

[15] Yan C., Li Q., Sun Q., Yang L., Liu X. (2023). Promising Nanomedicines of Shikonin for Cancer Promising Nanomedicines of Shikonin for Cancer Therapy. Int. J. Nanomed..

[16] Liao P.-L., Lin C.-H., Li C.-H., Tsai C.-H., Ho J.-D., Chiou G.C.Y., Kang J.-J., Cheng Y.-W. (2017). Anti-inflammatory properties of shikonin contribute to improved early-stage diabetic retinopathy. Sci. Rep..

[17] Song Y., Ding Q., Hao Y., Cui B., Ding C., Gao F. (2023). Pharmacological Effects of Shikonin and Its Potential in Skin Repair: A Review. Molecules.

[18] Han J., Chen T.X., Branford-White C.J., Zhu L.M. (2009). Electrospun shikonin-loaded PCL/PTMC composite fiber mats with potential biomedical applications. Int. J. Pharm..

[19] Arampatzis A.S., Kontogiannopoulos K.N., Theodoridis K., Aggelidou E., Rat A., Willems A., Tsivintzelis I., Papageorgiou V.P., Kritis A., Assimopoulou A.N. (2021). Electrospun wound dressings containing bioactive natural products: Physico-chemical characterization and biological assessment. Biomater. Res..

[20] Arampatzis A.S., Giannakoula K., Kontogiannopoulos K.N., Theodoridis K., Aggelidou E., Rat A., Kampasakali E., Willems A., Christofilos D., Kritis A. (2021). Novel electrospun poly-hydroxybutyrate scaffolds as carriers for the wound healing agents alkannins and shikonins. Regen. Biomater..

[21] Ramalho M.B., Durães A.F.S., Silvério F.O., Pinho G.P. (2020). Determination of three cresol isomers in sewage sludge by solid-liquid extraction with low temperature purification and gas chromatography-mass spectrometry. J. Environ. Sci. Health B.

[22] Rowe R.C., Sheskey P.J., Owen S.C. (2005). Handbook of Pharmaceutical Excipients.

[23] Prestinaci F., Pezzotti P., Pantosti A. (2015). Antimicrobial resistance: A global multifaceted phenomenon. Pathog. Glob. Health.

[24] Liu J.X., Yang L., Wan Y., Zhao W.E., Li S.W. (2024). Small Molecules Accelerate Skin Wound Healing: Shikonin Efficacy and Mechanism of Action in Mice. Int. J. Morphol..

[25] Hegab H., Tariq M., Syed N.A., Rizvi G. (2020). Towards Analysis and Optimization of Electrospun PVP (Polyvinylpyrrolidone) Nanofibers. Adv. Polym. Technol..

[26] Alshaya H.A., Alfahad A.J., Alsulaihem F.M., Aodah A.H., Alshehri A.A., Almughem F.A., Alfassam H.A., Aldossary A.M., Halwani A.A., Bukhary H.A. (2022). Fast-Dissolving Nifedipine and Atorvastatin Calcium Electrospun Nanofibers as a Potential Buccal Delivery System. Pharmaceutics.

[27] Alzahrani D., Alsulami K., Alsulaihem F.M., Bakr A., Booq R.Y., Alfahad A.J., Aodah A.H., Alsudir S., Fathaddin A., Alyamani E.J. (2024). Dual Drug-Loaded Coaxial Nanofiber Dressings for the Treatment of Diabetic Foot Ulcer. Int. J. Nanomed..

[28] Fu J., You L., Sun D., Zhang L., Zhao J., Li P. (2024). Heliyon Shikonin-loaded PLGA nanoparticles: A promising strategy for psoriasis treatment. Heliyon.

[29] Ameh E.S. (2019). A review of basic crystallography and x-ray diffraction applications. Int. J. Adv. Manuf. Technol..

[30] Barani H., Khorashadizadeh M., Haseloer A., Klein A. (2020). Characterization and Release Behavior of a Thiosemicarbazone from Electrospun Polyvinyl Alcohol Core-Shell Nanofibers. Polymers.

[31] Kontogiannopoulos K.N., Assimopoulou A.N., Tsivintzelis I., Panayiotou C., Papageorgiou V.P. (2011). Electrospun fiber mats containing shikonin and derivatives with potential biomedical applications. Int. J. Pharm..

[32] Sriyanti I., Edikresnha D., Rahma A., Munir M., Rachmawati H., Khairurrijal K. (2018). Mangosteen pericarp extract embedded in electrospun PVP nanofiber mats: Physicochemical properties and release mechanism of α-mangostin. Int. J. Nanomed..

[33] Fan C., Xie Y., Dong Y., Su Y., Upton Z. (2015). Investigating the potential of Shikonin as a novel hypertrophic scar treatment. J. Biomed. Sci..

[34] Yeung S., Lan W., Huang C., Lin C., Chan C., Chang M., Jeng J. (2002). Scavenging property of three cresol isomers against H_2_O_2_, hypochlorite, superoxide and hydroxyl radicals. Food Chem. Toxicol..

[35] Chang M., Chang H., Chan C., Yeung S. (2014). p-Cresol Affects Reactive Oxygen Species Generation, Cell Cycle Arrest, Cytotoxicity and Inflammation/Atherosclerosis-Related Modulators Production in Endothelial Cells and Mononuclear Cells. PLoS ONE.

[36] Al Zahrani N.A., Alshabibi M.A., Bakr A.A., Almughem F.A., Alshehri A.A., Al-Ghamdi H.A., Tawfik E.A., Damiati L.A. (2025). Molecular Hybrids of Thiazolidinone: Bridging Redox Modulation and Cancer Therapy. Int. J. Mol. Sci..

[37] Shu G., Xu D., Zhang W., Zhao X., Li H., Xu F., Yin L., Peng X., Fu H., Chang L.-J. (2022). Preparation of shikonin liposome and evaluation of its in vitro antibacterial and in vivo infected wound healing activity. Phytomedicine.

[38] Kaur K., Sharma R., Singh A., Attri S., Arora S., Kaur S., Bedi N. (2022). Pharmacological and analytical aspects of alkannin / shikonin and their derivatives: An update from 2008 to 2022. Chin. Herb. Med..

[39] Qiu H.-Y., Wang P.-F., Wang Z.-Z., Luo Y.-L., Hu D.-Q., Qi J.-L., Lu G.-H., Pang Y.-J., Yang R.-W., Zhu H.-L. (2016). Shikonin derivatives as inhibitors of tyrosyl-tRNA synthetase: Design, synthesis and biological evaluation. RSC Adv..

[40] Andújar I., Ríos J.L., Giner R.M., Recio M.C. (2013). Pharmacological properties of shikonin—A review of literature since 2002. Planta Med..

[41] Wan Y., Wang X., Zhang P., Zhang M., Kou M., Shi C., Peng X., Wang X. (2021). Control of Foodborne Staphylococcus aureus by Shikonin, a Natural Extract. Foods.

[42] Shin S. (2005). In Vitro Effects of Essential Oils from *Ostericum koreanum* against Antibiotic-Resistant *Salmonella* spp.. Arch. Pharm. Res..

[43] Drug Bank Cresol 2015. https://go.drugbank.com/drugs/DB11143.

[44] Bagheri F., Tahvilian R., Karimi N., Chalabi M., Azami M. (2018). Shikonin production by callus culture of onosma bulbotrichom as active pharmaceutical ingredient. Iran. J. Pharm. Res..

[45] Lee J.G., Shin S.-Y., Shin H.-J., Huh Y., Lee S.J., Kim D.-H., Lee S., Kim Y.-O., Han S.B., Lee J. (2012). Determination of three preservatives, cresol, chlorocresol and benzethonium, in drugs by high performance liquid chromatography-ultraviolet (HPLC-UV) detection. J. Pharm. Investig..

[46] Albreht A., Vovk I., Simonovska B. (2012). Addition of β-lactoglobulin produces water-soluble shikonin. J. Agric. Food Chem..

[47] O’Toole S.A., Sheppard B.L., McGuinness E.P.J., Gleeson N.C., Yoneda M., Bonnar J. (2003). The MTS assay as an indicator of chemosensitivity/resistance in malignant gynaecological tumours. Cancer Detect. Prev..

[48] (2018). Clinical and Laboratory Standards Institute Methods for Dilution Antimicrobial Susceptibility Tests for Bacteria That Grow Aerobically Standard, Approval CDM-A.

[49] Pannakal S.T., Prasad A., Phadke S., Sanyal A., Butti S., Khodr A., Morain C., Agnaou R., Shariff R., Benazzouz A. (2025). Acnocure, a Synergistic Anti-Microbial and Anti-Inflammatory Combination of Thymol and Curcuma Turmerones, Formulation and Time-Kill Studies Against *C. acnes*. Cosmetics.

