# Supplementary material for: Coaxial Electrospun Nanofibers of Shikonin and Cresol as Antibacterial Wound Dressing
Source: Pharmaceuticals (Basel). 2025 Oct 30;18(11):1642. doi: 10.3390/ph18111642 (PMC12655277; doi:10.3390/ph18111642)
Supplement: Supplementary file 1 [file pharmaceuticals-18-01642-s001.zip › pharmaceuticals-3838571-supplementary.pdf]

Supplementary Materials Section

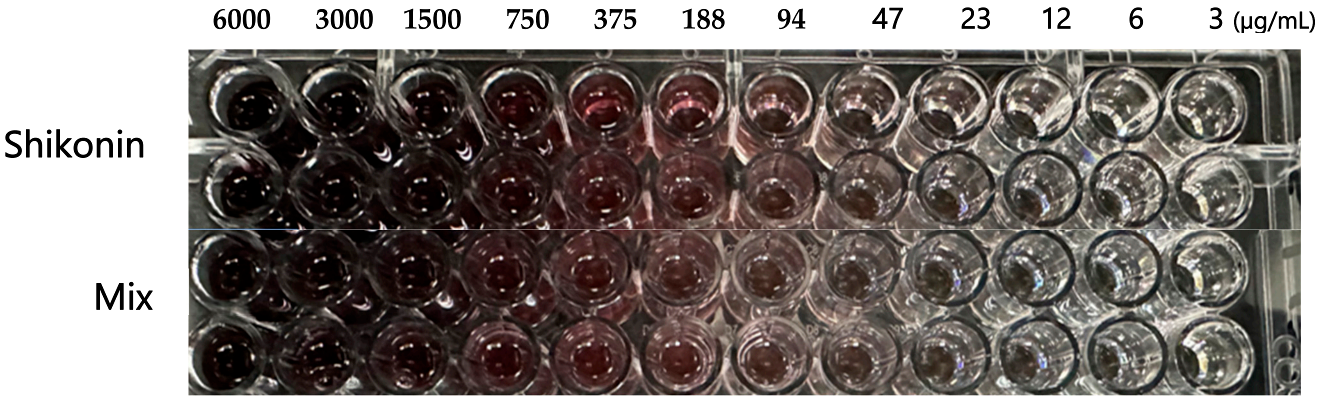

Figure S1. The 96-well plate represents the (MIC) of the blank plate for shi and the mix (shi mixed with cre 1:1) without any bacteria.

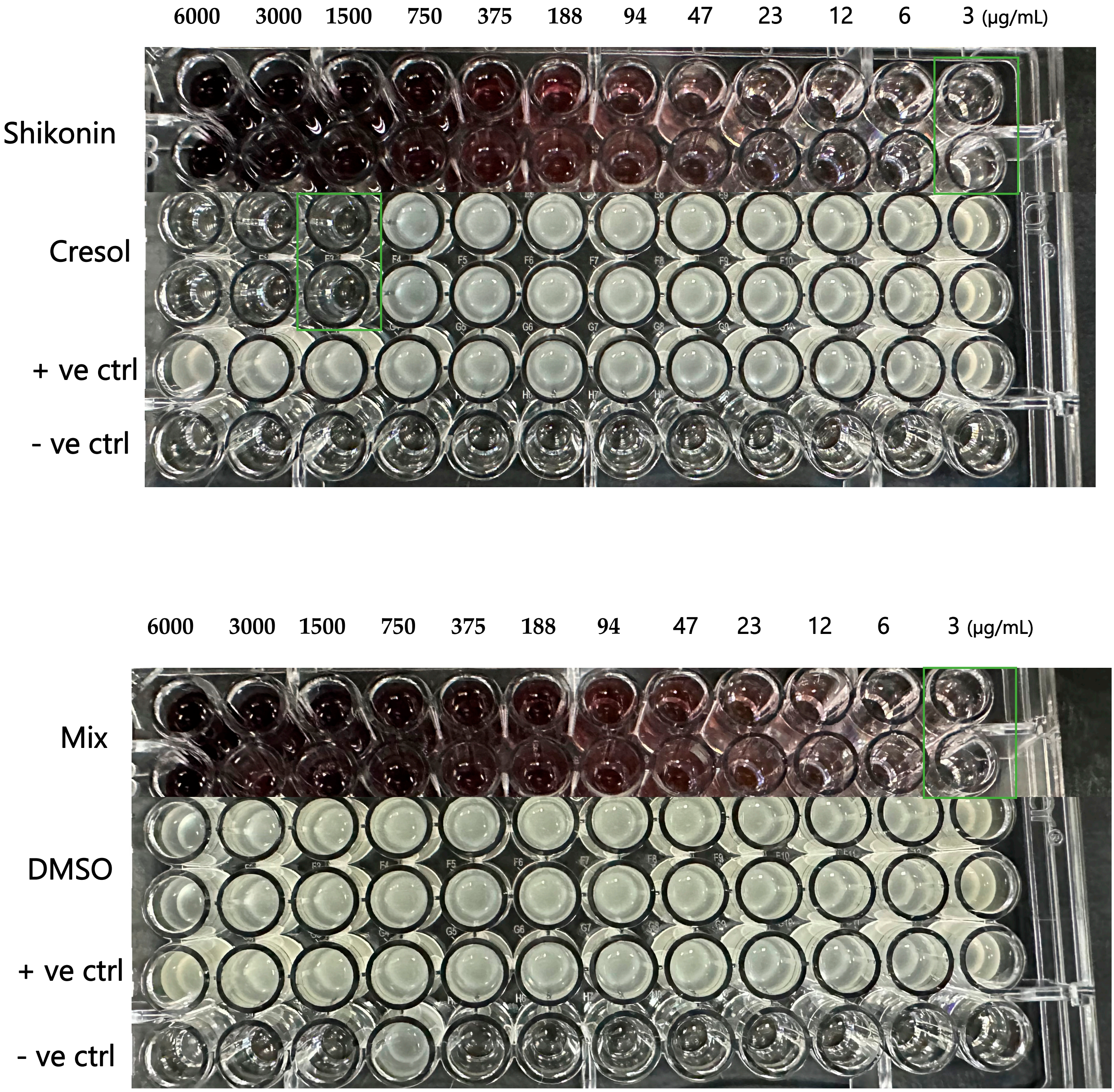

Figure S2. The MIC test against *S. aureus* (ATCC 29213). The green square is considered the MIC, which showed a clear well. Shi and the combination were at 3 μg/mL, and cre was at 1500 μg/mL.

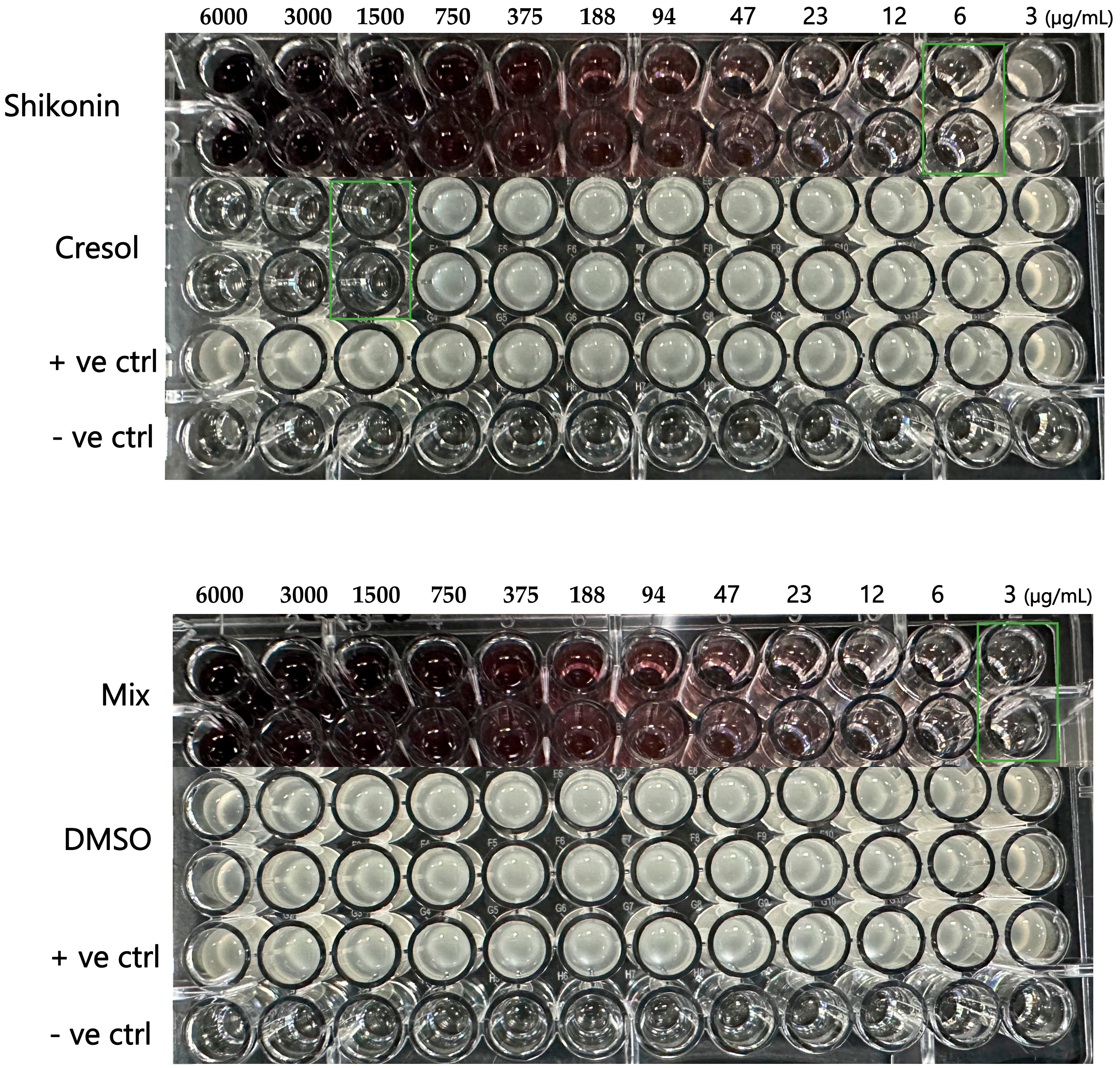

Figure S3. The MIC test against *MRSA* (ATCC 43300) as a clinical isolate. The green square is considered the MIC, which showed a clear well. Shi was at 6 μg/mL, cre was at 1500 μg/mL, and the combination was at 3 μg/mL.

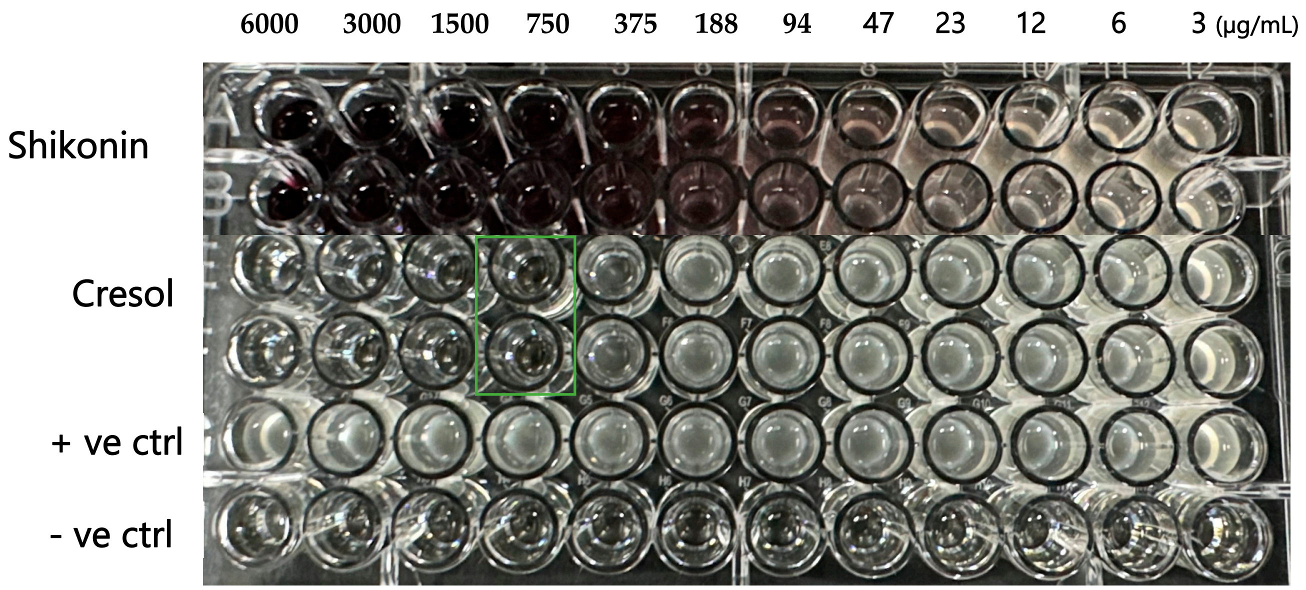

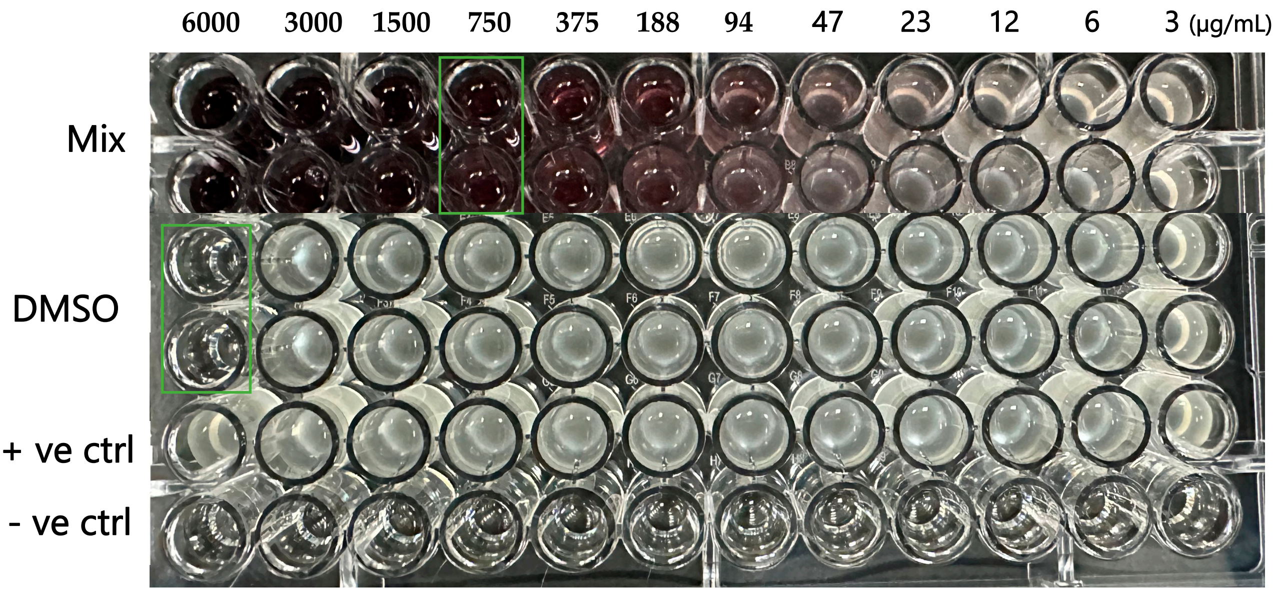

Figure S4. The MIC test against *E. coli* (ATCC 25922). The green square is considered the MIC, which showed a clear well. Shi has no inhibition, the cre was 750 μg/mL, and the combination was 750 μg/mL.

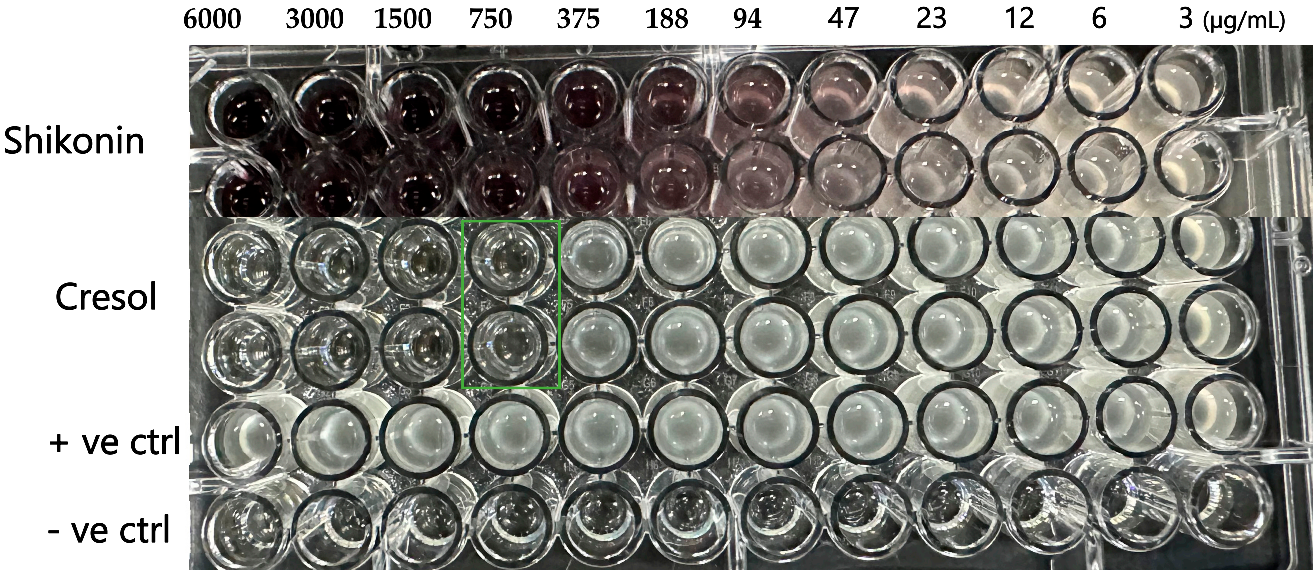

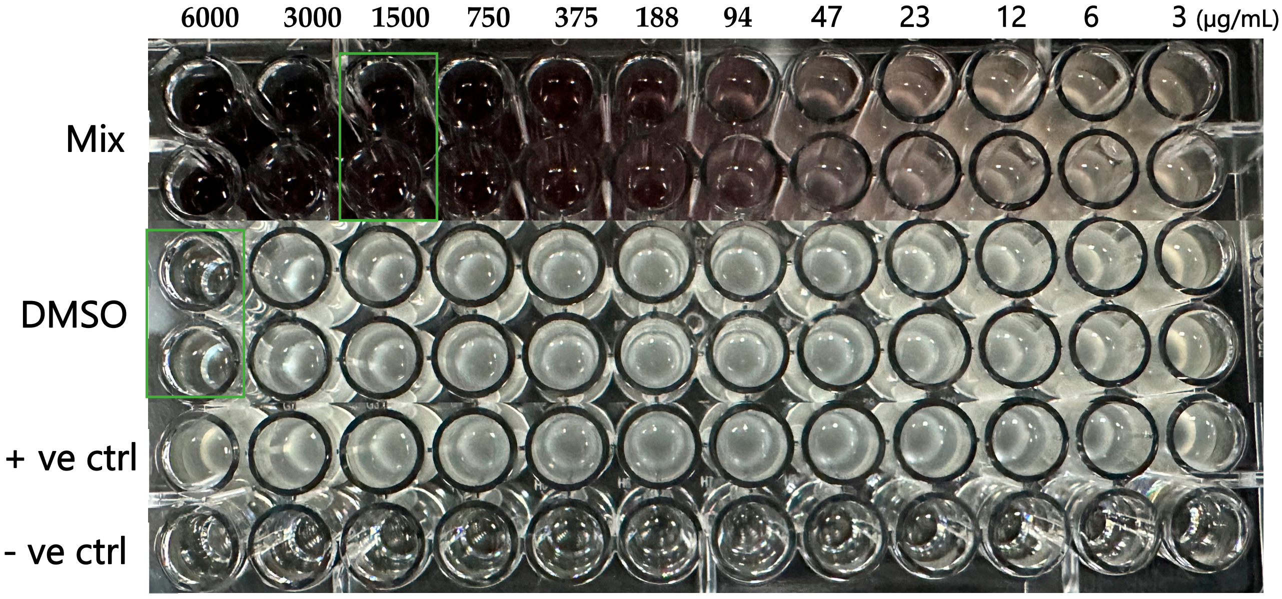

Figure S5. The MIC test against *E. coli* (isolate 1060) as a clinical isolate. The green square is considered the MIC, shi has no inhibition, cre was 750 µg/mL, and the combination was 1500 µg/mL.

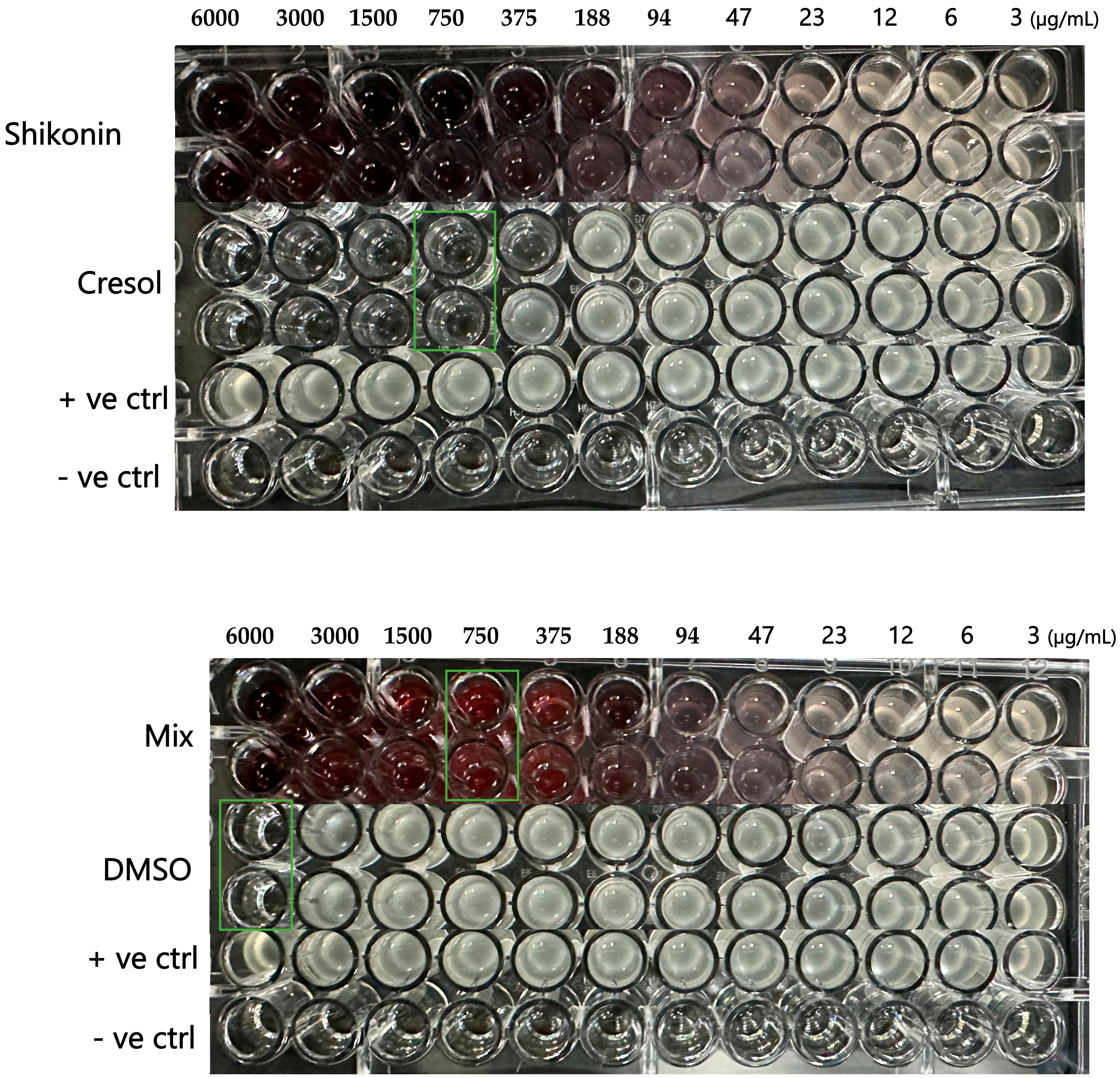

Figure S6. The MIC test against *A. baumannii* (ATCC BAA 747). The green square is considered the MIC, which showed a clear well. Shi has no inhibition, and the cre was 750 µg/mL, and the combination was 750 µg/mL.

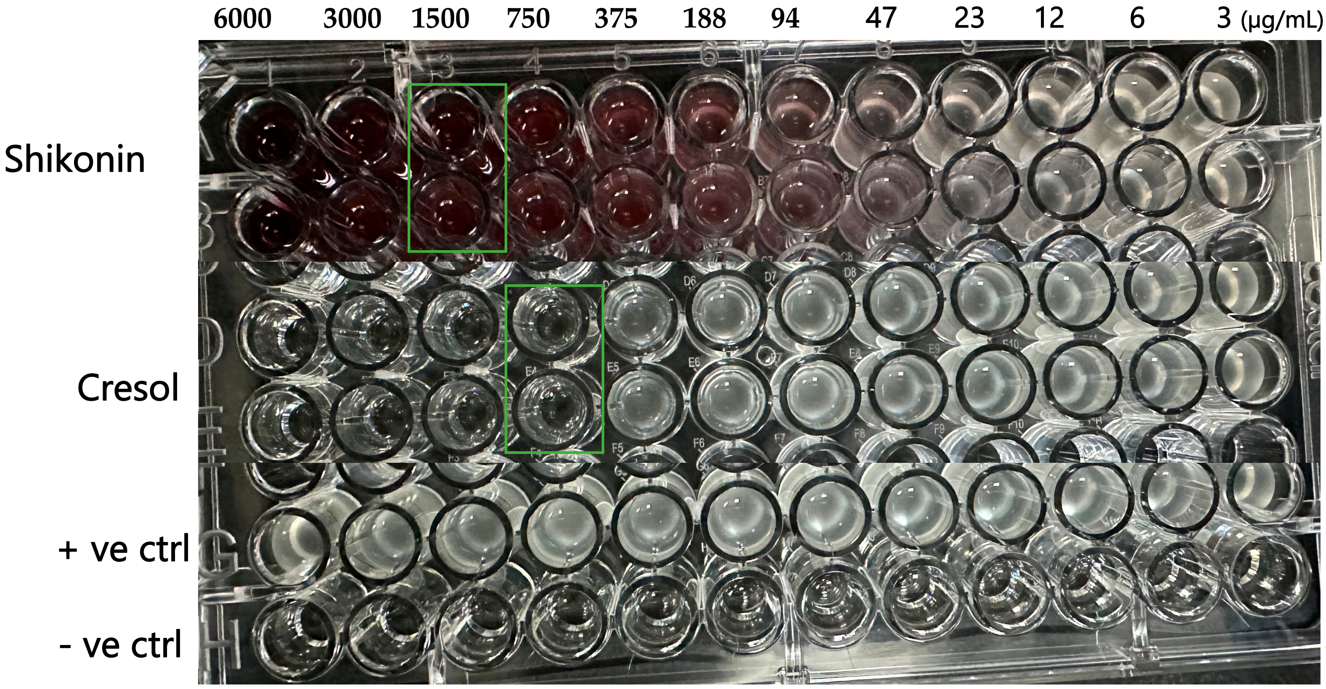

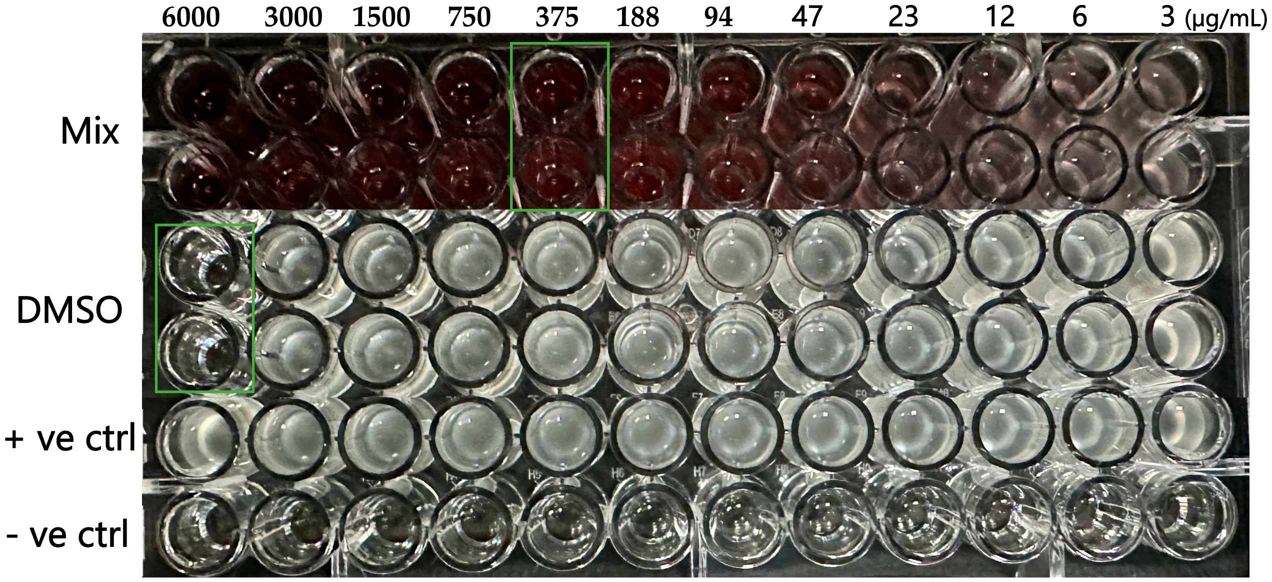

Figure S7. The (MIC) test against *A. baumannii* (isolate 3034). The green square is considered the MIC, which showed a clear well. shi was 1500 µg/mL, cre was 750 µg/mL, and the combination was 375 µg/mL.

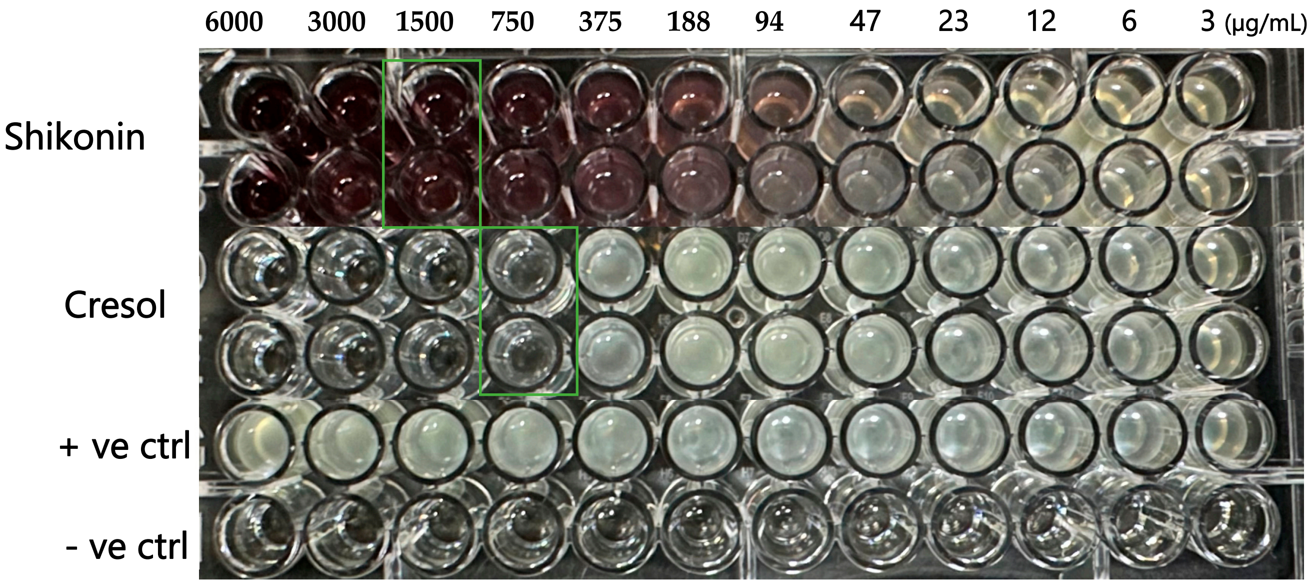

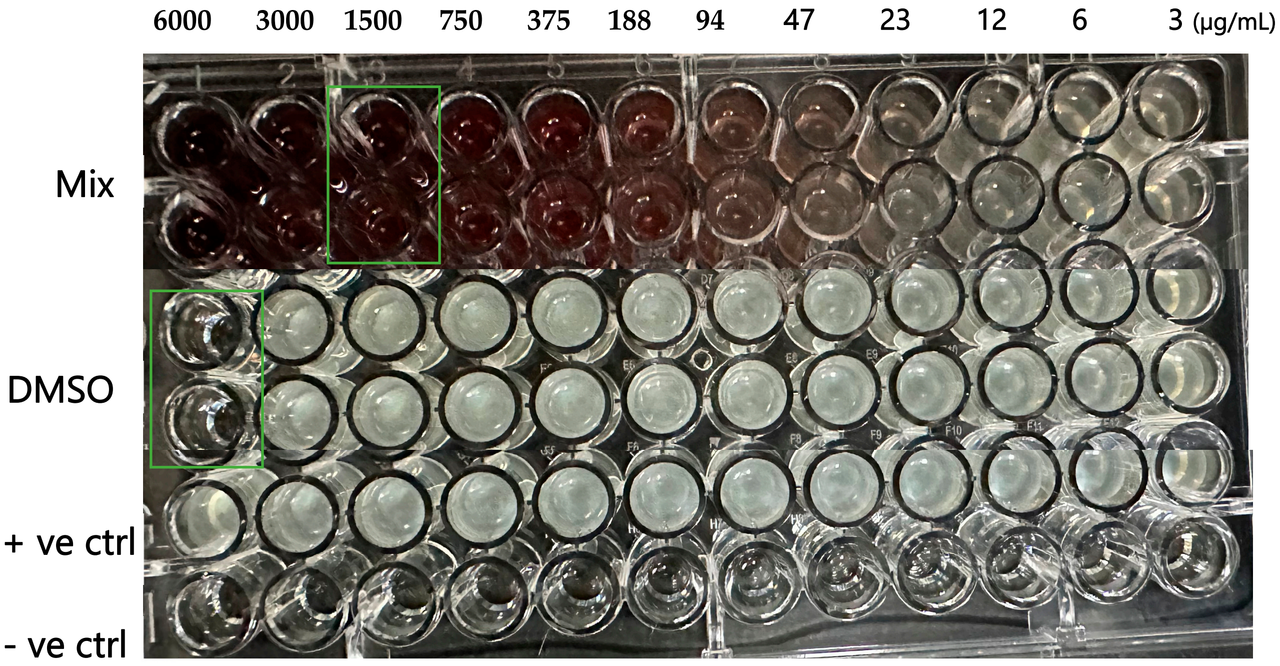

Figure S8. The MIC test against *P. aeruginosa* (ATCC 27853). The green square is considered the MIC, which showed a clear well. Shi was 1500 µg/mL, cre was at 750 µg/mL. And the combination was at 1500 µg/mL.

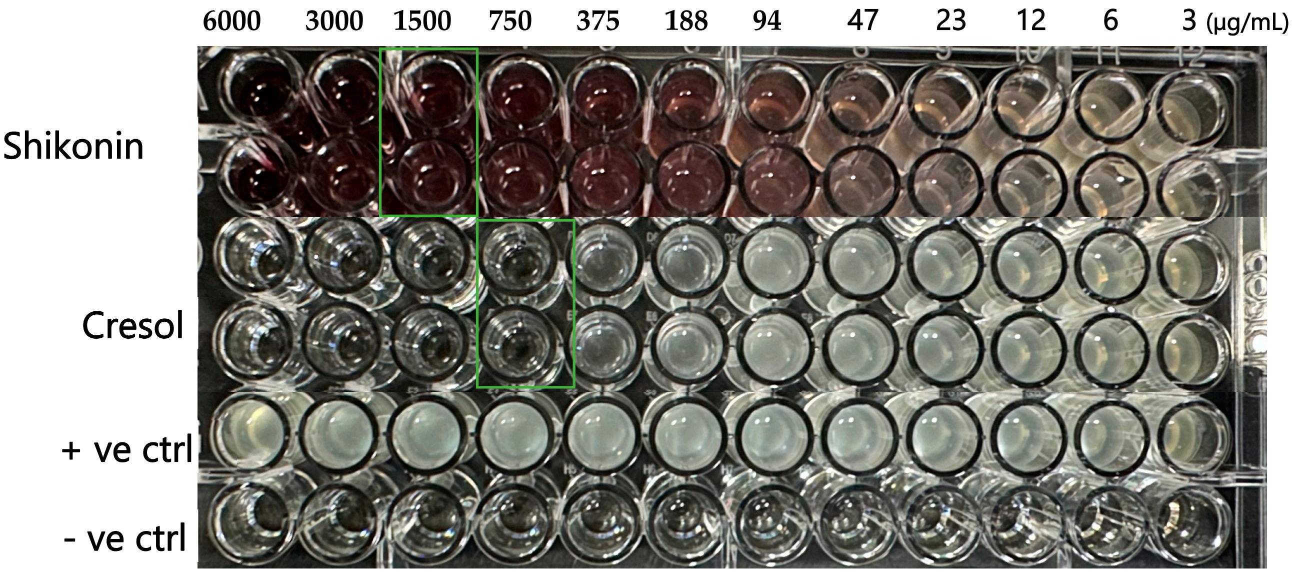

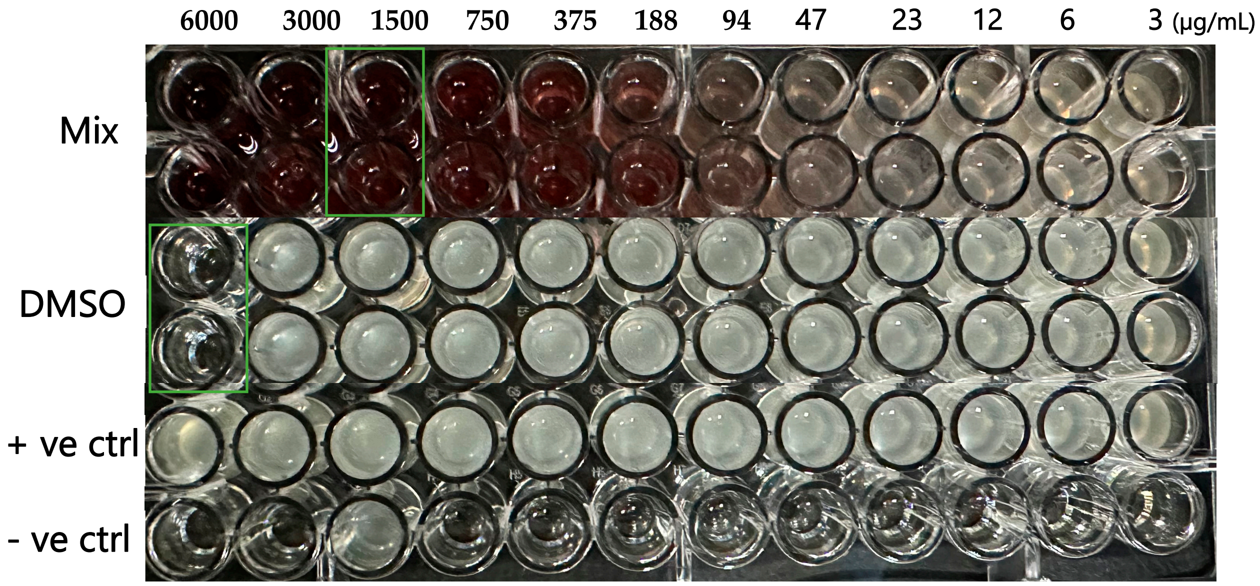

Figure S9. The MIC test against *P. aeruginosa* (isolate 7067) as a clinical isolate. The green square is considered the MIC, which showed a clear well. Shi was 1500 μg/mL, cre was at 750 μg/mL, and the combination was 1500 μg/mL.
